# RLS-associated MEIS transcription factors control distinct processes in human neural stem cells

**DOI:** 10.1038/s41598-024-80266-9

**Published:** 2024-11-22

**Authors:** Volker Kittke, Chen Zhao, Daniel D. Lam, Philip Harrer, Wojciech Krezel, Barbara Schormair, Konrad Oexle, Juliane Winkelmann

**Affiliations:** 1 Institute of Neurogenomics, Helmholtz Munich, Neuherberg, Germany; 2 Institute of Human Genetics, Klinikum rechts der Isar, School of Medicine, Technical University of Munich, Munich, Germany; 3 DZPG (German Center for Mental Health), Munich, Germany; 4 Global Computational Biology & Digital Sciences, Boehringer Ingelheim Pharma GmbH & Co. KG, Biberach an der Riß, Germany; 5 Institut de Génétique et de Biologie Moléculaire et Cellulaire (IGBMC), Illkirch, France; 6 Munich Cluster for Systems Neurology, SyNergy, Munich, Germany

**Keywords:** Developmental biology, Development of the nervous system, Genetics of the nervous system, Transcriptomics, CRISPR-Cas9 genome editing, Neural stem cells, Immunoprecipitation, Gene expression analysis, Movement disorders

## Abstract

*MEIS1* and *MEIS2* encode highly conserved homeodomain transcription factors crucial for developmental processes in a wide range of tissues, including the brain. They can execute redundant functions when co-expressed in the same cell types, but their roles during early stages of neural differentiation have not been systematically compared. By separate knockout and overexpression of *MEIS1* and *MEIS2* in human neural stem cells, we find they control specific sets of target genes, associated with distinct biological processes. Integration of DNA binding sites with differential transcriptomics implicates MEIS1 to co-regulate gene expression by interaction with transcription factors of the SOX and FOX families. *MEIS1* harbors the strongest risk factor for restless legs syndrome (RLS). Our data suggest that MEIS1 can directly regulate the RLS-associated genes *NTNG1*, *MDGA1* and *DACH1*, constituting new approaches to study the elusive pathomechanism or RLS.

**Supplementary Information:**

The online version contains supplementary material available at 10.1038/s41598-024-80266-9.

## Introduction

Restless legs syndrome (RLS) is one of the most common neurological disorders with a prevalence of 5–10% in the population of European ancestry^1^. Symptoms manifest as an urge to move the legs, primarily during the evening and night. Movement can temporarily relieve the symptoms. Severe RLS is found in 2–3% of the general population^2^, which require treatment, usually including dopaminergics, α_2_δ ligands or opioids, pointing towards an indirect involvement of the dopaminergic system^3^.

The etiology however is mostly unclear, with a multitude of genetic and environmental factors contributing to the disease. Despite RLS being most prevalent in the adult population, genome-wide association studies have implicated neurogenesis in the development of the disorder^4^. Pathway enrichment analyses of RLS-associated genes^5 ^indicated early embryonic neural development is a vulnerable phase in the generation of RLS disposition, while the specific affected cell types remain unresolved at present. Therefore, we conducted our study on a common representative of neurogenesis, that is, human neural stem cells (hNSC). hNSC can differentiate towards excitatory, inhibitory and glial lineages, and also partly mirror transcription factor occupancy during fetal brain development^6^. Our cell line is euploid and does not express HOX-genes, thus more closely mirrors conditions in forebrain, midbrain and cerebellum^7^, as compared to common neuroblastoma cell lines.

The strongest RLS association signal identified by GWAs is within a linkage disequilibrium (LD) block in intron 8 of *MEIS1*containing several highly conserved non-coding elements^4,5,8,9^. The function of *MEIS1* and these elements in neurodevelopment and disease remains to be clarified, particularly in relation to RLS. Direct interaction of the RLS-associated intronic region with the *MEIS1 *promoter has been demonstrated in different neural cell types^10^, and may explain reduced MEIS1 expression reported in brain tissue of RLS patients carrying the *MEIS1 *RLS risk variant^11,12^. *MEIS2*, a highly conserved paralog of *MEIS1*, is also located within a region associated with risk of RLS, although the association is disproportionately lower compared to the *MEIS1*^4^.

*MEIS1* and *MEIS2 *encode TALE homeodomain transcription factors (TF)^13^, sharing 85% protein sequence identity and bind the same DNA motifs^14^. Both MEIS1 and MEIS2 control various non-neural developmental processes^15–19^. In addition, both genes also regulate neural development in striatum, midbrain, cerebellum, retina and sympathetic neurons as well adult neurogenesis in the olfactory bulb^14,20–24^, and are also expressed in neural stem cells^25,26^. Functional redundancy, or at least partial compensation of the loss of either paralog, has been observed in limb, neural crest and eye development^14,17,18,27–29^, and in pathological conditions including various tumors^30–33^. Despite partly overlapping expression patterns in the developing forebrain^34^, the redundancy of *MEIS1* and *MEIS2* in the CNS has not been analyzed directly and remains poorly understood.

Using in vitro cultured human neural stem cells (hNSC), we aimed to elucidate the target genes and networks directly controlled by *MEIS1* and *MEIS2* during neural development, by ways of knockout and overexpression (OE) of these genes. For the first time, we directly compare the effect of *MEIS1* and *MEIS2* OE and KO on the transcriptome in a neurodevelopmental context. Furthermore, we investigated the MEIS1 gene-regulatory mechanisms using ChIP-seq to identify downstream factors involved in neurodevelopmental processes and thereby, potentially, also in the development of RLS.

## Results

### MEIS1 and MEIS2 do not affect each other’s expression

**Fig. 1:**
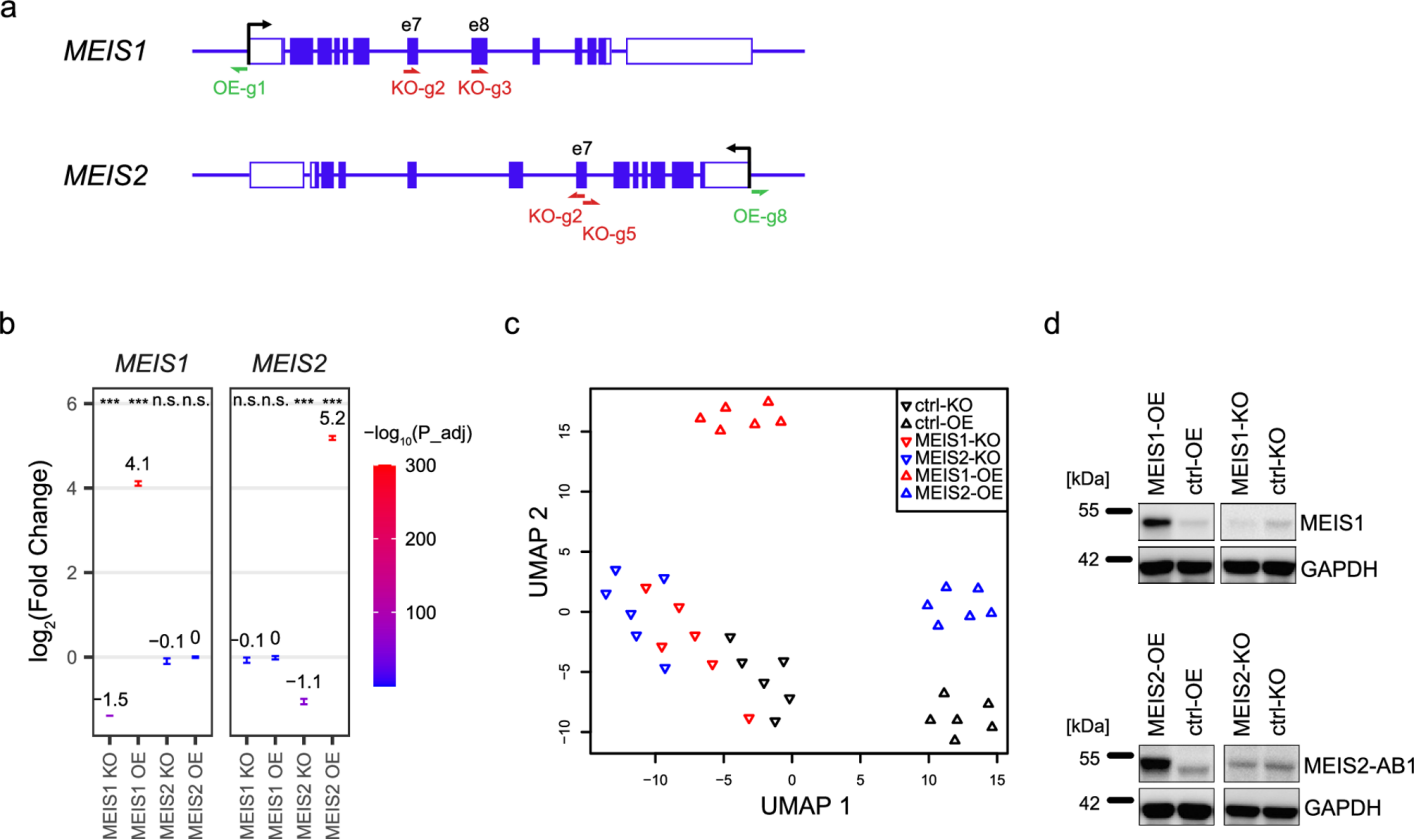
MEIS knockout and overexpression in hNSC. (**a**), Schematic representation of CRISPR guide positions at *MEIS1* and *MEIS2* gene loci. Exons correspond to isoform a of MEIS1 and MEIS2 proteins. **(b)**, effects of CRISPR-KO and CRISPR-activation on mRNA expression of *MEIS1* and *MEIS2*. log2(fold changes) are reported from pairwise differential gene expression by DESeq2, relative to controls. Numbers indicate mean log2(fold change), error bars represent mean ± s.e.m. *** adjusted P-value < 0.001. (**c)**, UMAP visualization of replicates highlights expression differences between treatment groups. (**d)**, representative western blots of hNSC protein lysates after CRISPR treatment compared to respective controls. Original blots are presented in Supplementary Fig. 7.

We started by manipulating the expression of *MEIS1* and *MEIS2 *either by CRISPR/Cas9-mediated knockout (KO) or overexpression (OE) via CRISPR-activation^35–37^ in cultured human neural stem cells (hNSC). KO and OE were performed separately for *MEIS1* and *MEIS2*, with non-targeting sgRNAs serving as controls (*n* = 6 per condition). Cells were transduced with lentiviral vectors encoding for sgRNAs and CRISPR/Cas9 or the CRISPR-activation machinery (Fig. 1a) and harvested 48 h after transduction, in order to detect direct effects of KO and OE and to identify direct target genes. We found no changes in cell density or morphology specific to any treatment condition (Supplementary Fig. S1). In parallel, RNA and protein were extracted from the same samples.

We measured gene expression by RNA-seq and initially performed pairwise differential gene expression analysis for each condition (MEIS1-OE, MEIS1-KO, MEIS2-OE, MEIS2-KO) against the respective control (ctrl-OE or ctrl-KO) using DEseq2^38^ (Supplementary Table S1). *MEIS1* and *MEIS2* overexpression resulted in strong increase of mRNA expression (log_2_(fold-change) of 4.2 and 5.2, respectively) while KO reduced mRNA expression by 65% and 53%, respectively. KO and OE were also specific to either *MEIS1* or *MEIS2*, without affecting the expression of the other paralog (Fig. 1b).

Similar expression changes were also detected on the protein level by western blotting, with MEIS1 strongly increased after OE, while a decrease after KO was difficult to detect due to the low base expression level (Fig. 1d & Supplementary Fig. S2). MEIS2-OE resulted in a strong increase of protein expression, with the band running slightly higher than the control bands (Fig. 1d & Supplementary Fig. S2). We suspected that this band corresponds to isoform MEIS2A (401 aa), while the band visible in control cells also incorporates MEIS1A (390aa), which cannot be distinguished due to the small size difference. We therefore tested the specificity of two anti-MEIS2 and the anti-MEIS1 antibody against overexpressed MEIS1, MEIS2 and MEIS1-GFP proteins. While anti-MEIS1 only reacted to MEIS1 and MEIS1-GFP, we observed strong reactivity to all proteins for both anti-MEIS2 antibodies (Supplementary Fig. S2), explaining the discrepancy of bands in MEIS2-OE. The effect of MEIS2-KO could therefore not be detected (Supplementary Fig. S2). Another antibody, specific to MEIS1A and MEIS2A isoforms, proved higher sensitivity, and thus was able to resolve two proteins (Supplementary Fig. S2).

Potential off-targets of the CRISPR/Cas9 sgRNA were determined using CRISPOR^39^, and considered for further evaluation at a CFD score > 0.1 and if located either in the exon or intron of a gene. Of the potential off-target genes, only *SMOC1*, *KIAA1644 and RAPGEF3* were differentially expressed when compared to ctrl-KO. All genes turned out to be affected by both *MEIS1* and *MEIS2* and therefore cannot be considered off-targets of a specific guide sequence (Supplementary Table S3).

We assessed the effect of *MEIS1* and *MEIS2 *KO and OE on global gene expression by uniform manifold approximation and projection (UMAP^40,41^), and found that samples clustered mainly according to treatment condition (Fig. 1b). Transfection of the CRISPR-systems themselves already conveyed a strong effect, visible by the distinct clustering of the ctrl-OE and ctrl-KO samples. In general, similarity was higher among knockout conditions than among OE conditions, indicating a stronger effect conveyed by *MEIS* overexpression compared to *MEIS* knockout.

### MEIS1 and MEIS2 targets are involved in distinct developmental processes

We used DESeq2^38^ to identify transcriptome-wide significant differential expressions (DE) that resulted from the CRISPR-induced changes of either *MEIS1* or *MEIS2* levels and thus indicated their target genes. In our model we directly contrasted the effects of OE and KO, yielding a combined measure of the expression level of a *MEIS *gene (see Methods^38^). Thereby, we increased specificity of the target gene identification, since random expression changes of a gene that point in the same direction in OE and KO cancel each other out, and non-specific effects caused by either of the CRISPR treatments are compensated for (see Methods section for further details). We henceforth refer to genes whose expression is positively correlated with *MEIS1* expression, i.e. genes upregulated in MEIS1-OE and/or downregulated in MEIS1-KO, as MEIS1-upregulated genes (MEIS1-UreG). Analogously, genes are termed MEIS1-downregulated (MEIS1-DreG) if their expression is negatively correlated with *MEIS1* expression. The same classification applies to MEIS2 targets.

**Fig. 2:**
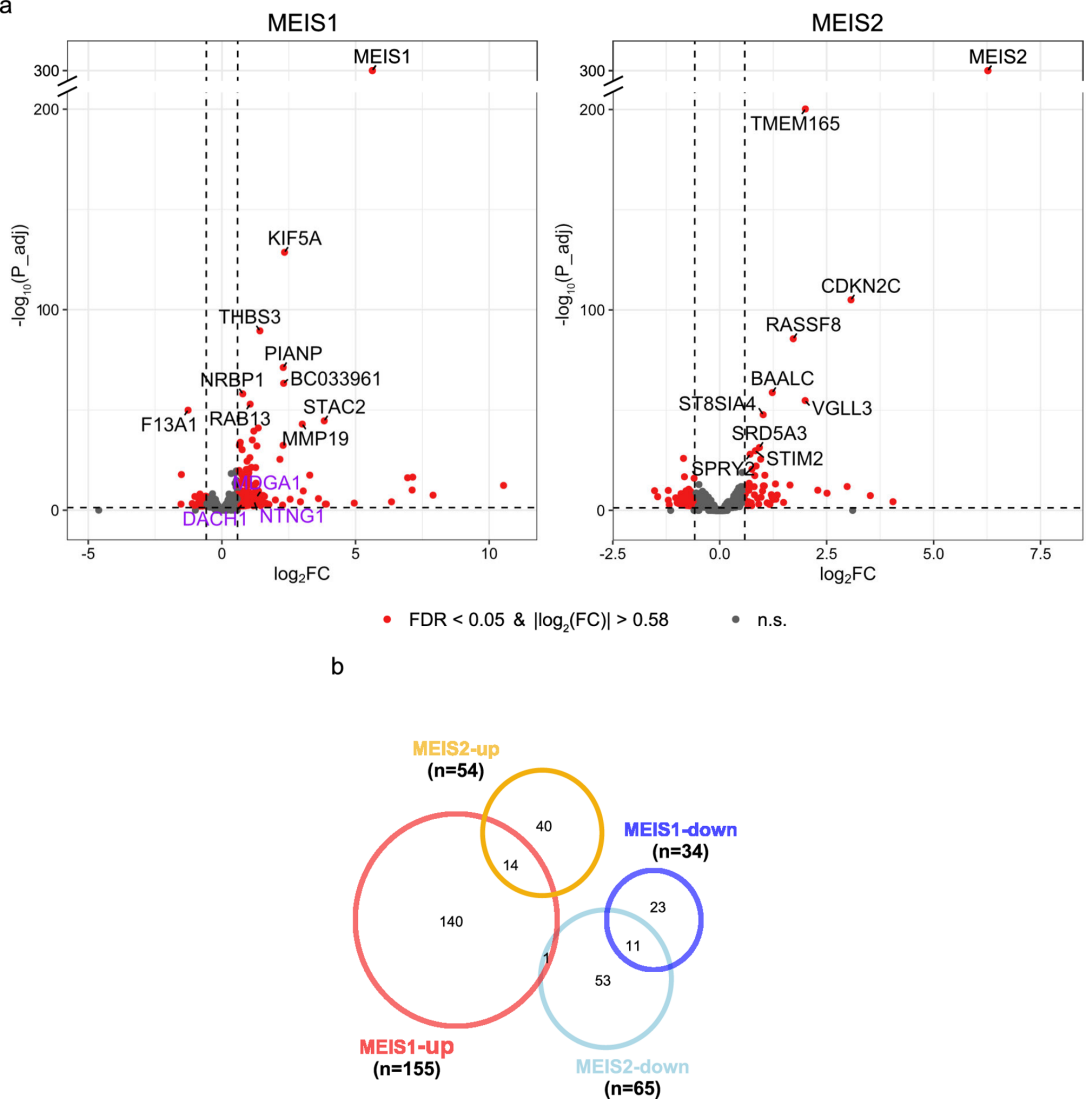
MEIS1 and MEIS2 control distinct sets of target genes. (**a**), volcano plots of differential expression by MEIS1 and MEIS2 CRISPR treatment, using a combined model of KO and OE effects. log_2_FC refers to expression changes of OE contrasted with the effect of KO, each normalized to their respective control. Differentially expressed, RLS-associated genes are highlighted in purple. Dashed lines indicate cutoffs: FDR < 0.05, |log_2_(foldchange)| > 0.58. (**b)**, Venn diagram outlining overlap of differentially expressed MEIS1 and MEIS2 target genes.

Thus, we identified 155 MEIS1-UreG, 34 MEIS1-DreG, 54 MEIS2-UreG and 65 MEIS2-DreG genes (adjusted *P* < 0.05, |log_2_(fold-change)| > 0.58, Fig. 2a, b., Supplementary Table S2). The overlap between the target genes of MEIS1 and MEIS2 was highly significant (Fisher’s exact test, OR = 48.3 [23.7–93.2] with *P* = 4.8*10^−18^ for upregulated genes, OR = 172.3 [72.3–387.3] with *P* = 5.6*10^−20^ for downregulated genes).

Due to their shared DNA binding motif and high protein sequence similarity, we expected MEIS1 and MEIS2 to regulate the same target genes under consistent experimental design and threshold settings. To assess the expected overlap of Differentially Expressed Genes (DEGs) under this assumption, we employed bootstrap resampling, calculated the pairwise relative overlaps (Jaccard index J, see Methods) of bootstrapped samples of MEIS1 DEGs only and of bootstrapped samples of MEIS2 DEGs only, and thus derived the expected overlaps with confidence intervals (CI, Table 1). For upregulated DEGs we derived J_MEIS1_ = 0.511, 95% CI [0.339–0.717], based on MEIS1-UreG and J_MEIS2_ = 0.493, 95% CI [0.268, 0.673], based on MEIS2-UreG. Both expected overlaps differed significantly from the observed overlap of J = 0.072, 95% CI [0.031, 0.094] between MEIS1-UreG and MEIS2-UreG (*P* = 2.48*10^−6^ and *P* = 4.49*10^−5^, respectively; one-sample Z-score test).

**Table 1:** Jaccard indices from bootstrap analysis comparing target genes of MEIS1 and MEIS2. Bootstrap resampling analysis was used to determine expected overlap of MEIS1 or MEIS2 target genes under the assumption that both target the same genes. Jaccard Indices (Jaccard similarity coefficients) measure the number of genes in the intersection of two gene lists, divided by the number of target genes in the union of the gene lists. P values are computed from a one-sample Z-score test, comparing expected overlap among bootstrapped samples with observed overlap between MEIS1 and MEIS2 targets.

Jaccard similarity coefficient [95% confidence interval]						
	Observed	Expected				
	J_MEIS1 vs. MEIS2_	J_MEIS1_	*P* (J_MEIS1_ vs. J_MEIS1 vs. MEIS2_)	J_MEIS2_	*P* (J_MEIS1_ vs. J_MEIS1 vs. MEIS2_)	O/E ratio
Up	0.072	0.511 [0.339–0.717]	2.48E-06	0.493[0.268–0.673]	4.49E-05	14.3%
Down	0.126	0.226 [0.033–0.364]	0.246	0.303[0.120–0.474]	0.051	47.6%
All	0.092	0.414 [0.221–0.620]	6.37E-04	0.368[0.190–0.536]	1.20E-03	23.5%

For downregulated DEGs, the expected overlaps did not differ significantly from the observed overlap between MEIS1 and MEIS2. Notably, the expected overlap of all DEGs (J_MEIS1_ = 0.414, 95% CI [0.221, 0.620], J_MEIS2_ = 0.368, 95% CI [0.190, 0.536]) also differed significantly from the observed overlap (J_MEIS1_MEIS2_ = 0.092; *P* = 6.37*10^−4^ and *P* = 1.2*10^−3^, respectively). A stronger effect of MEIS1 and MEIS2 on UreG compared to DreG was also mirrored in the overall lower adjusted P-values of upregulated genes compared to downregulated genes (Fig. 2., median adjusted P-values: MEIS1-UreG = 2.06*10^−06^, MEIS1-DreG = 8.64*10^−04^, MEIS2-UreG = 1.38*10^−09^, MEIS2-DreG = 5.36*10^−05^).

In addition, we analyzed the correlation between Z-scores of the DEG analysis using a mixture linear model to evaluate overall target gene specificity. While significant, the correlation of overall gene expression changes induced by MEIS1, compared to MEIS2, was low when considering the high degree in homology of the two TFs (*P* = 3*10^−107^, R² = 0.348, Supplementary Fig. S3).

**Fig. 3:**
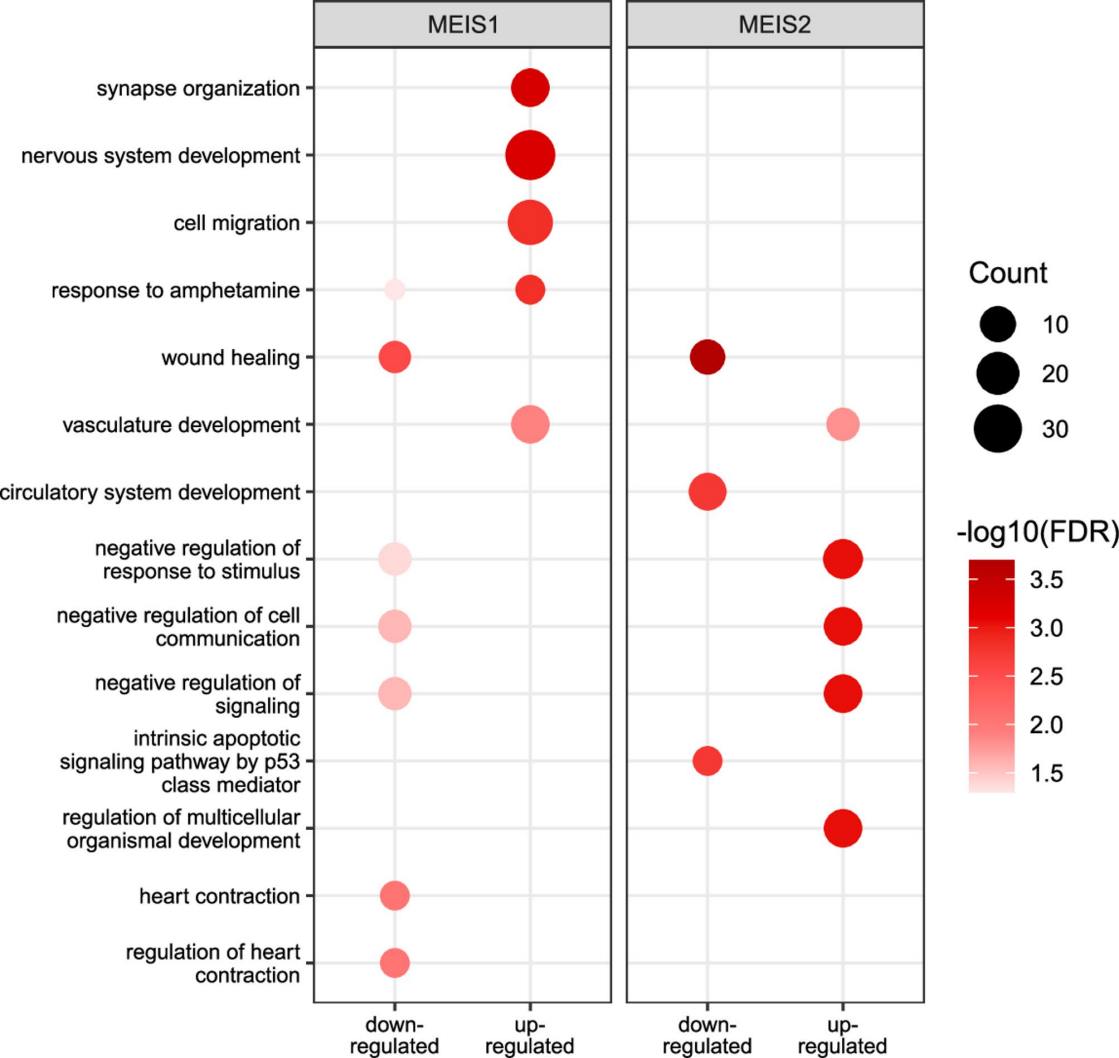
High level of pathway specificity for genes regulated by MEIS1 vs. MEIS2. Overrepresentation analysis showing enrichment of differentially upregulated or downregulated genes in biological processes from Gene Ontology. FDR cutoff = 0.05.

This dissimilarity indicated that MEIS1 and MEIS2 may regulate distinct gene sets and corresponding processes in hNSC. Hence, we performed overrepresentation analysis (ORA), using significantly up- and downregulated genes to test an enrichment in known terms of the Gene Ontology Biological Process database^42,43^. MEIS1-UreG were enriched in regulators for nervous system development, synapse organization and cell migration (Fig. 3). Genes involved in synapse organization encoded both neurotransmitter receptors (*GABRB3*) as well as cell adhesion proteins like thrombospondin2 (*THBS2/TSP2*) and neurexin 2 (*NRXN2*). Notably, we found MEIS1-UreG enriched in amphetamine response, a process involving dopamine signaling^44^. These genes included *PPP1R1B*/DARPP-32, expressed in dopamine-receptor positive neurons in the basal ganglia and cerebral cortex both during development and in the adult brain^45,46^.

MEIS1-DreG were related to non-neural processes like, wound healing, cell signaling as well as heart contraction, in line with *MEIS1*being implicated in PR-interval prolongation^47^.

Several gene sets enriched for MEIS2-regulated genes, including wound healing, vasculature development and signaling-associated processes, overlapped with gene sets enriched for MEIS1-regulated genes. (Fig. 3, Supplementary Table S4).

In gene sets that were enriched for target genes of both MEIS1 and MEIS2, we observed little overlap of genes (Supplementary Fig. S4), highlighting the difference between MEIS1 and MEIS2 effects described above and indicating that MEIS1 and MEIS2 can employ different gene regulatory networks.

We thus arrived at a concept of independent and specific functions for MEIS1 and MEIS2 in hNSC, due to strong differences in their targets and regulated processes, as well as lack of mutual regulation.

### Inspection of MEIS binding sites

We investigated the mechanism of transcriptional regulation by MEIS in hNSC, by determining its binding sites via chromatin-immunoprecipitation sequencing (ChIP-seq) in untreated cells, using a combination of anti-MEIS1/2A and anti-MEIS1A/B antibodies^48^.

We identified 5169 MEIS binding sites in the genome, of which 12% were located within ± 5 kb of a transcriptional start site (Supplementary Table S5, Supplementary Fig. S5), confirming previous findings of MEIS predominantly binding regions distal to TSS^14,20,48^. Among these, we detected binding sites in the promoters and intronic regions of both *MEIS1* and *MEIS2* (Supplementary Fig. S6), potentially allowing mutual or autoregulation of transcription.

**Fig. 4:**
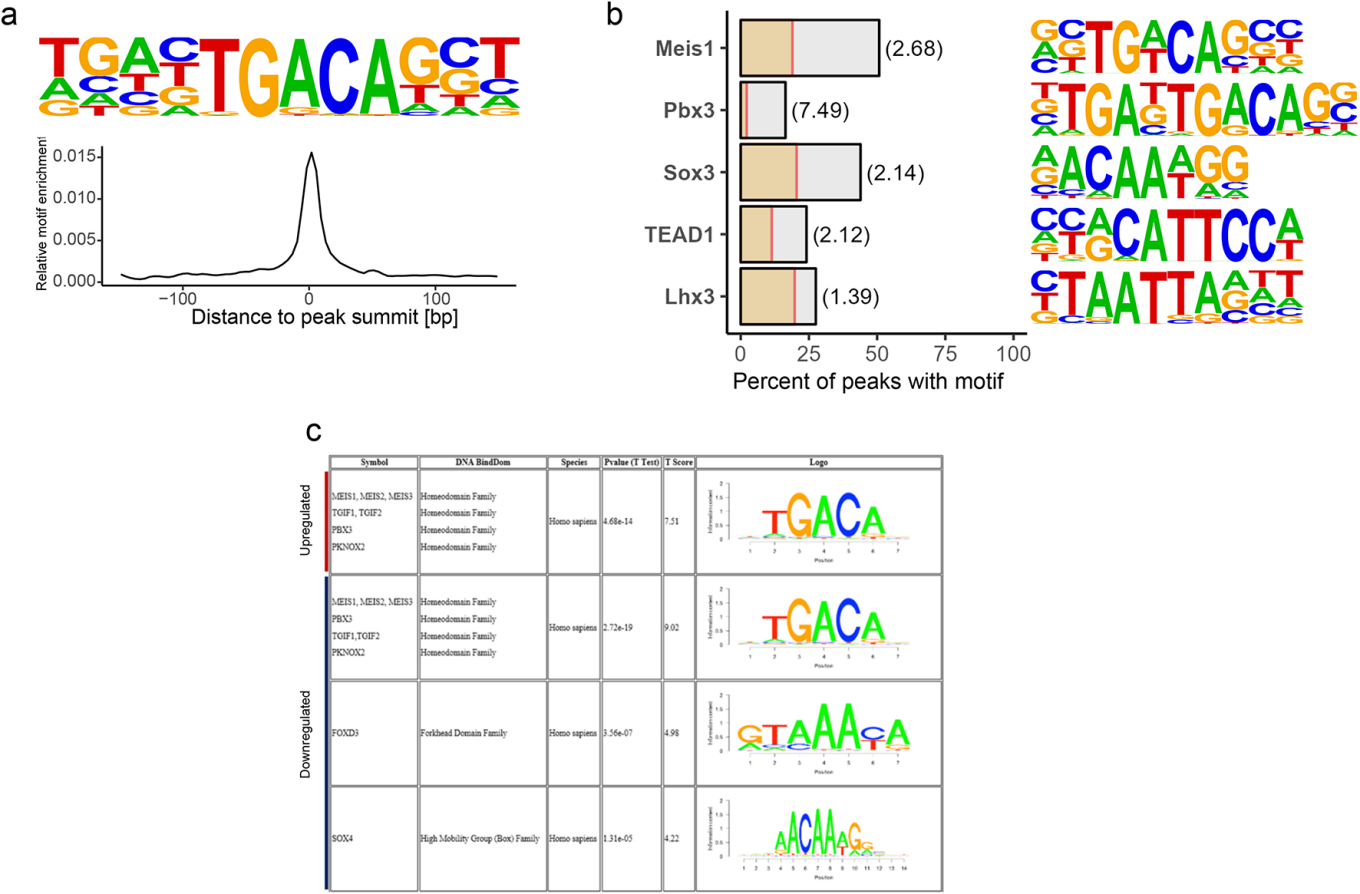
Binding sites of MEIS1 in hNSC. (**a**), MEIS1 consensus motif is most enriched in *de novo* motif discovery. Position weight matrix (PWM) and motif distribution across all peaks relative to peak summits. (**b)**, Frequency of peaks containing known DNA binding motifs (grey bars). Yellow bars show frequency in GC-matched background sequences. Enrichment is given in parenthesis. (**c)**, Motifs associated with up- or downregulation of target genes, determined via Binding and Expression Target Analysis (BETA).

To verify the efficacy of the ChIP-seq experiment, and to identify potential co-binding partners of MEIS1, we performed de novo motif detection via HOMER within all MEIS binding sites^49^ and found the known MEIS consensus binding motif TGACAG strongly enriched at the peak centers (Fig. 4a, Supplementary Fig. S5). HOMER also detected a 12 bp motif containing the TAATT motif, bound by several homeodomain families^50 ^(Supplementary Fig. S5), which displayed a clear bimodal distribution, indicating the presence of a possible co-transcription factor^51,52^. We performed motif enrichment analysis of known TF motifs within MEIS peaks, to identify putative co-regulators of MEIS and detected an enrichment of DNA motifs of the TALE transcription factor PBX3, SOX3, TEAD1 and LHX3 (Fig. 4b). PBX family proteins interact directly with MEIS proteins via specialized interaction domains, thereby regulating activity, protein stability and intracellular localization^53^. SRY-Box (SOX) proteins share a conserved binding motif and are key drivers of proliferation in neural stem cells^54,55^.

### FOX and SOX DNA binding motifs are associated with negative regulation by MEIS1

Binding of a TF alone does not warrant a regulatory effect but may require specific co-regulators. Therefore, we investigated the relation between MEIS binding sites and RNA expression of nearby genes using Binding and Expression Target Analysis (BETA), which integrates gene expression changes in all genes with transcription factor binding sites and thus associates peaks with nearby genes^56^. Thus, we identified 119 upregulated and 29 downregulated MEIS1 target genes that were associated with a MEIS binding site, suggesting a direct regulation either via a distal regulatory element or by direct promoter interaction. To achieve higher test power, we included all DEG with FDR < 0.05 for TF function prediction, showing MEIS1 was able to both activate and repress its direct target genes (Supplementary Fig. S5, Supplementary Table S6). To identify potential co-regulators of MEIS1, we performed separate motif enrichment analysis with either up- or downregulating binding sites and found the MEIS consensus binding motif (TGACAG) motif associated with both activating and repressive function (Fig. 4c). In addition, the SOX4 and FOXD3 transcription factor motifs were associated with downregulated genes.

### MEIS1 upregulates RLS candidate genes involved in neurogenesis

*MEIS1 *has been speculated to control the expression of other RLS associated genes^12,57^. We therefore compared target genes of MEIS1 with 37 potential RLS-associated genes determined in our previous GWAS^4^. As a control, we compared RLS-associated genes with targets of MEIS2, which has been suggested to compensate reduced MEIS1 levels in RLS patients^58^.

We found three of these genes among MEIS1 targets, *MDGA1*,* NTNG1*, and *DACH1*, while no RLS-associated genes were detected among MEIS2 targets. All of these were predicted to be directly regulated by MEIS1, based on the combined analysis of ChIP-seq and RNA-seq (Supplementary Table S6).

**Fig. 5:**
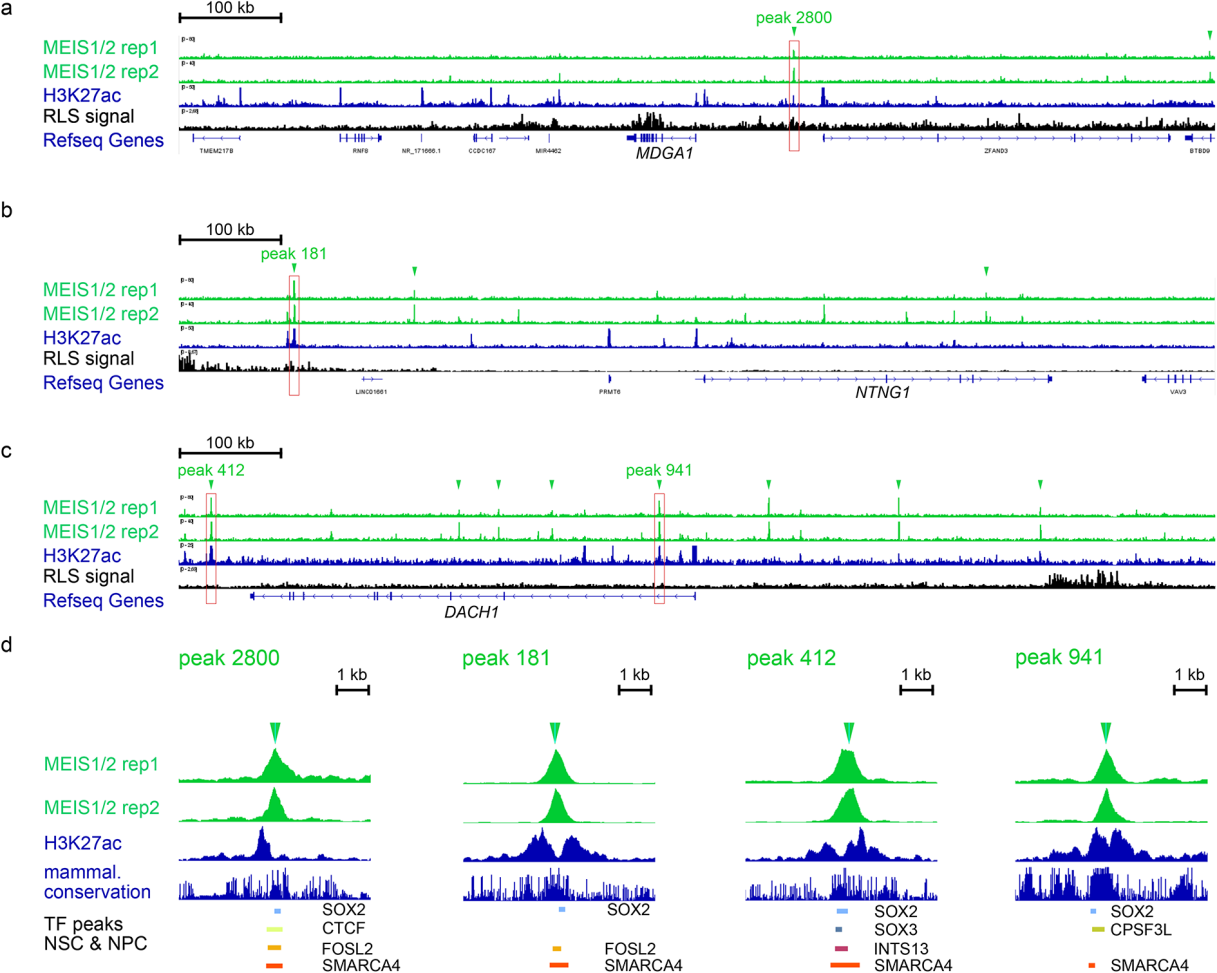
MEIS1/2 directly regulate RLS-associated genes by binding to their promoters or nearby regulatory regions. Binding landscape of MEIS1/2 around (**a)**, *MDGA1*, (**b)**, *NTNG1*, (**c)**, *DACH1*. (**d) **MEIS1/2 peaks overlapping with H3K27ac are shown in detail, along with mammalian conservation and TF binding sites in neural stem & progenitor cells. Two replicates of MEIS1/2 ChIP-Seq are shown, peaks were determined using IDR. H3K27-acetylation data was previously generated in ESC-derived hNSC^60^. RLS SNP association shows Z-scores of GWAS summary statistics for each SNP^4^. Arrowheads indicate called MEIS1/2 peaks.

We performed close inspection of the MEIS1/2 binding landscape around each gene. For *MDGA1 *we found a MEIS1/2 binding site in a predicted enhancer, 92 kb upstream of the TSS^59^ (Fig. 5). We found one intragenic binding site within *NTNG1.*Interestingly, one of the overall strongest MEIS1 binding sites was found 386 kb upstream of NTNG1, in a region with high genetic RLS association, based on our previous GWAS^4^. For DACH1 we found four intronic and several upstream MEIS1 binding sites, including predicted enhancers at −195 kb, + 35 kb + 139 kb, + 228 kb relative to the transcriptional start site.

We filtered MEIS1/2 binding sites in the vicinity of *MDGA1*, *NTNG1* and *DACH1 *for overlap with H3K27ac, marking active enhancers^60^. These peaks were analyzed for overlap with binding sites of 18 TF in human neural stem cells and neural progenitor cells using the ChIP-Atlas^61^(Fig. 5d). Apart from binding sites of ubiquitous TF CTCF, FOSL2 (AP-1 complex) and SMARCA4/BRG1 (SWI/SNF complex), we found that all active MEIS1/2 binding sites overlap with those of SOX2, which is in line with the enrichment of SOX-family motif in all MEIS1/2 binding sites (Fig. 4).

## Discussion

We investigated the function of *MEIS1* and *MEIS2 *in human neural stem cells (hNSC) in vitro. They encode TFs with widely overlapping expression patterns, and hold similar functions in various tissues^14,17,18,27,28,62^. To date, their interplay in the central nervous system has remained elusive. Here, we report that MEIS1 and MEIS2 control differing sets of genes and pathways in hNSC, and that they do not regulate each other’s expression. In addition, we identified direct MEIS1 target genes which are known to be associated with RLS and therefore are candidates for mediating the strong effect of *MEIS1* on RLS pathogenesis.

We detected high target specificity of MEIS1 and MEIS2, with 93% of the upregulated and 86% of the downregulated genes being specific for only one paralog. Correspondingly, gene ontology analysis revealed involvement in differing regulatory pathways.

Considering the high level of conservation of the MEIS1 and MEIS2 protein sequences and their identical homeodomain, required for DNA binding^48,63^, this finding is rather unexpected. Several studies described regulation of identical processes by MEIS1 and MEIS2, including Delgado et al. who found both MEIS1 and MEIS2 being essential in limb development^18^. Regulation of the same developmental processes implies, however, that MEIS1 and MEIS2 target predominantly the same genes. Our opposite finding of paralog-specific target genes in hNSC therefore suggests that the functional overlap of MEIS1 and MEIS2 depends on cell type, that is, on the prevailing regulatory network.

Cell type specific target gene selection can be achieved by interaction with varying sets of co-transcription factors^64–66^. MEIS proteins predominantly form heterodimers with PBX family members via highly specific homology domains^4,48,67–71^. Through PBX, MEIS can also form trimeric MEIS-PBX-HOX complexes^72^. The unstructured C-terminal domain allows direct interaction with HOX and non-HOX TF^73–76^. Therefore, tissue-specific expression of co-TF can enable MEIS to bind different regulatory regions in a cell-type dependent manner.

On the other hand, it has not been investigated how MEIS1 and MEIS2 can regulate different targets in the same cell type, despite sharing 84% sequence identity and binding the same DNA motif. One study found target specificity of paralog TFs is mostly driven by protein variation outside the DNA binding domains (DBD), since interchanging the DBD between paralogs only slightly affected target preference^77^. This would be in line with the DBD (homeodomain) of MEIS1 and MEIS2 being 99% conserved, and the C-terminal domain displaying more sequence variation between MEIS1 and MEIS2^78^, possibly affecting binding affinity with other proteins. Furthermore, paralog TFs can compete for DNA binding motifs in a concentration-dependent manner^79^, enabling different functions in the same cell types. For example, loss of MEF2D increases MEF2A occupancy of binding sites normally bound by MEF2D in the mouse cerebellum^80^. Moreover, in vitro binding assays revealed slight variations in DNA motif preferences by paralogs, independent of co-transcription factors^81^. Similar investigations are required for MEIS1 and MEIS2 to determine if there are indeed different motif preferences, which may have remained elusive in previous studies. Systematic comparison of MEIS1 and MEIS2 binding sites, and study of the individual interactomes are required in the future to delineate target gene selection processes in different cell types.

While we found no indication of MEIS1 and MEIS2 regulating the same processes in hNSC, functional redundancy of the two paralogs has been observed in the limb and eye^14,17,62^, though underlying mechanisms are unclear.

Mutual regulation between the genes was described by Marcos et al., where KO of *Meis1* caused upregulation of *Meis2, *resulting only in mild defects of lens development in the mouse eye^20^ and raising the question how *Meis2* upregulation could be achieved. While in hNSC, *MEIS1* and *MEIS2 *do not influence each other’s mRNA expression, we did detect MEIS1/2 binding sites in both genes, which also had been observed in previous studies^20,48,62^(Supplementary Fig. S6), and is a general characteristic of many other TFs. Stabilization of expression levels by mutual as well as autoregulatory transcriptional inhibition can determine cell lineage commitment and can vary throughout developmental stages^82^. Autoregulation may also be important for the role in acquisition and maintenance of neuronal identity^83,84^as has been suggested for MEIS1^24^ but less so at the stem cell stage. Moreover, MEIS1-HOXA9 dependent autoregulation of *MEIS1*is believed to result in elevated expression, and thus accelerated disease progression in acute leukemia^85,86^. Furthermore, mechanisms for the compensation of knockouts by homologous genes can also involve nonsense-mediated mRNA decay and subsequent binding of mRNA snippets to similar DNA sequences^87–89^. KO of *Meis2* results in a similar lens phenotype, but the effect on *Meis1*expression was not evaluated^62^. However, complete knockout of both *Meis1* and *Meis2 *in the eye causes earlier and more extensive developmental defects than single knockouts of either gene^14,20,62^. Similarly, loss of either *Meis* paralog during mouse skeletal development leads to minor growth defects, while loss of both paralogs results in complete limb agenesis, indicative of an additive effect^17,18^.

Cell type specificity of target gene selection and compensatory mechanisms can both be determined by interaction with varying sets of co-transcription factors, which may also underlie the disproportionately stronger RLS-association *of MEIS1* compared to *MEIS2*.

*MEIS1 *being the strongest genetic risk factor in RLS, we searched for putative mechanisms of MEIS1 contribution to the elusive pathomechanism. Identifying possible interaction partners, we combined motif analysis within MEIS binding sites with MEIS1 target genes and found the SOX and FOX transcription factor families to be likely co-regulators of MEIS1 in hNSC. SOX2 is vital for proliferation of neural stem cells in the ventricular zone of the lateral ganglionic eminence (LGE), giving rise to the striatum during embryonic development^54,90,91^. FOXP1 and FOXP2 are required for differentiation and normal function of striatal spiny projection neurons (SPN)^92–95^. In these cell types, MEIS1 is co-expressed with SOX2, FOXP1 and FOXP2^26,34 ^and decreased interaction of MEIS1 with either SOX2, FOXP1 or FOXP2 thus could affect striatal development, connectivity and function. Dopamine-receptor type 2 SPN (D2-SPN) displayed decreased dopamine binding affinity in RLS patients^96,97^, while another study found decreased functional connectivity of dopaminergic pathways^98^. Unilateral striatal lesions in mice resulted in increased activity, a key characteristic of RLS mouse models^99,100^. Similarly, D2-SPN-specific knockout of *Ppp1r1b (*DARPP-32), a key regulator of dopaminergic signaling in all SPN^101,102^, resulted in increased locomotion in mice^103^. We found *PPP1R1B* (DARPP-32*)*, highly upregulated by MEIS1 in hNSC. In contrast, *PPP1R1B *was unaffected by MEIS2, despite MEIS2 being expressed in developing and adult SPN, and being essential for their differentiation from neural progenitors^34,104–106^. This indicates that MEIS1 and MEIS2 may control different functions in the striatum. Notably, somatic conditional deletion of MEIS1 in D2-SPN did not recapitulate the RLS phenotype observed by *Meis1* haploinsufficiency in *Meis1*^+/−^mice^107^ hinting at a more complex mechanism, involving multiple risk factors.

Furthermore, altered dopamine signaling has been described in multiple brain regions in RLS patients, and consequently synaptic plasticity may be decreased^89,97,108,109^. We found regulators of synaptic organization upregulated by MEIS1, among which the RLS-associated genes *NTNG1*, *MDGA1* and *DACH1 *were identified^4,110–112^. The strong RLS association of *MEIS1* may partly be founded in controlling the expression of other RLS associated genes, whereby aberrant *MEIS1*expression would directly affect the expression of these genes^57,113^.

In summary, we delineate independent and varying regulatory roles of MEIS1 and MEIS2 in human neural stem cells, contrary to their nature in many other tissues. We identified possible co-regulators of MEIS1 and specific MEIS1 target genes linked to RLS and present further developmental effectors potentially involved in RLS.

Further investigation of neural cell types expressing MEIS1, and especially showing regulatory activity of RLS-associated regions will be required to provide information on the RLS pathomechanism.

While this study is limited to examining direct transcriptional effects of *MEIS* dysregulation, further reasearch will have to include the temporal dynamics of gene regulation by MEIS1 and MEIS2. Moreover, long-term effects of *MEIS1* and *MEIS2* perturbation should be studied on the phenotypic level.

## Methods

### Cell culture & lentivirus generation

Guide RNA sequences for CRISPR-activation and CRISPR-KO (**see Resources**) were designed using CRISpick^110,111^ and cloned into LentiCRISPRv2 and sgRNA(MS2)_puro optimized backbone.

Lentivirus was generated using the psPAX2 packaging and pMD2.G envelope plasmids along with one of the transfer plasmids encoding the target gene for integration. Lenti-X 293T cells (Takara Bio™, 632180) were seeded 24 h prior to transfection onto 10 cm culture dishes at 6 Mio cells per dish. 0.7 pmol pMD2.G, 1.3 pmol µg psPAX2, 1.6 pmol target vector were mixed with 600 µl Opti-MEM (Gibco™, 11058021), 48 µl polyethyleneimine (PEI, 1 mg/ml, Polysciences™ 24765-1), and incubated for 10–20 min at room temperature before adding the mixture to the culture dish. Cells were incubated for 16 h at 37 °C and 5% CO_2_, growth media was replaced and cells cultivated for another 2 days. Culture media containing lentivirus was harvested 72 h after transfection, centrifuged at 1200 xg for 5 min at 4 °C. Supernatants were filtered with 0.45 μm ployethersulfone (PES) syringe filters. Viral particles were concentrated using the Lenti-X concentrator (Takara Bio™ 631232) accoding to the manufacturer’s protocol. Supernatant was carefully removed with a serological pipet and the lentiviral pellet was resuspended with DPBS and diluted to a total volume of 1 ml. Virus was stored at −80 °C.

Human Neural Stem Cells (hNSC) were purchased from Gibco™ (N7800-100), and cultured in 10 ml media in 10 cm dishes coated with Geltrex (Gibco™ A1413201) according to the manufacturer’s protocol. Cells were passaged at a ratio of 1:3 − 1:6 using Accutase (Sigma A6964).

For CRISPR-activation, 275 µl of each concentrated lentivirus sgRNA(MS2)_puro, lenti dCAS-VP64_Blast and lentiMPHv2 was applied per 10 cm dish. For CRISPR-KO, a total of 500 µl lentivirus lentiCRISPRv2 was applied. Virus targeting MEIS1 (MEIS1-g2 and MEIS1-g3) or MEIS2 (MEIS2-g2 and MEIS2-g5), were mixed at 1:1 ratio, respectively.

Media was changed after 18 h and cells harvested 48 h after viral transduction by dissociating with Accutase and washing once with DPBS. RNA was extracted using the RNeasy Mini Kit (Qiagen).

We analyzed the amount of protein and RNA in each sample as proxies of cell count, thereby allowing conclusions towards growth rate and cell survival. They indicated a small to moderate effect of the KO and OE compared to the respective controls, but this effect was not consistently significant across protein and RNA analyses, however (see Supplementary Fig. S2).

### Western blot analysis

Protein was isolated from in vitro cultured cells using ice-cold lysis buffer (150 mM NaCl, 1% Triton X-100, 50 mM Tris-Cl pH 8.0, 0.5% Na-Deoxycholate, 0.1% SDS, protease inhibitor) by agitating for 30 min at 4 °C. Lysates were centrifuged at 10,000 xg for 10 min at 4 °C, and supernatants transferred to protein lo-bind tubes. Protein concentration was determined by BSA standard curve, using the DC Protein Quantification Assay (Bio-Rad, 500 − 0116) according to the manufacturer’s instructions. Absorption was measured on a NanoDrop One (Thermo Fisher Scientific) at 750 nm.

Up to 30 µg total protein per sample was used for western blot, along with 10 µl ProSieve™ QuadColor™ Protein Marker (Lonza). Volume was adjusted to 15 µl with TBST before adding 3 µl 6x Lämmli Buffer (66 mM Tris-HCl, 10% SDS (w/v), 52% Glycerol (v/v), 100 mM DTT, 666 µg/ml Bromophenol blue) and boiling at 95 °C for 5 min.

Samples were loaded on 12% Mini-Protean TGX Gel (456–1095, Bio-Rad) in a Mini-Protean Tetra System (Bio-Rad). Gels were transferred semi-dry to nitrocellulose membranes using the Trans-Blot Turbo system (Bio-Rad) and visualized in a Fusion FX chemiluminescence system.

### RNAseq

RNA extraction from cryopreserved cells was performed with the RNeasy Kit (Qiagen) with on-column DNase treatment and elution in H2O. After RNA isolation, RNA integrity number (RIN) was measured using the Agilent 2100 Bioanalyzer system. RNAs with a RIN value > 7 were selected for mRNA sequencing (poly-A selected). The libraries were prepared using the TruSeq stranded mRNA Sample Preparation kit (Illumina), following the kit’s instructions. After a final QC, the libraries were sequenced in paired-end mode (2 × 100 bases) on the Novaseq6000 sequencer (Illumina) with a depth of ≥ 10 Million reads per sample.

Reads were aligned to the human genome (hg19) using the STAR (v 2.4.2a)^114^and were counted using HTSeq v0.6.0^115^.

### RNA-seq analysis

#### Differential gene expression analysis

DESeq2 v1.34.0 was used for differential gene expression analyses.

For each *MEIS1* and *MEIS2*, 6 CRISPR-KO (KO) and 6 CRISPR-activation (overexpression, OE) datasets were derived as well as the corresponding control datasets using non-targeting guide RNA (6 ctrl-KO and 6 ctrl-OE). The latter were used for correction of non-specific effects by experimental stress due to vector transfection and CRISPR-system activity.

To identify the regulated genes, we employed a contrast matrix in DESeq2 which subtracted the KO effect from the OE effect. This approach enabled us to discern effects that were consistent in upregulated and downregulated conditions, while filtering out genes that merely responded to experimental stress. Concretely, DESeq2 fitted and thus normalized the read counts separately in each of the six different treatments, MEIS1-OE, MEIS1-KO, MEIS2-OE, MEIS2-KO, ctrl-OE, and ctrl-KO. The regulatory effects of MEIS1 were then estimated by using a contrast that stated (MEIS1-OE – ctrl-OE) – (MEIS1-KO – ctrl-KO); the regulatory effects of MEIS2 were estimated accordingly. By including count data from both OE and KO to estimate the dispersion using DESeq2, we were able to correct the overall expression changes caused by the CRISPR-knockout or CRISPR-activation systems alone, and thereby reduce false positives due to treatment effects. The shrunken logFC was reported using the adaptive shrinkage estimator ashr v2.2–54. In total, 19,689 out of 28,516 genes were included in the analysis with at least 10 counts across all samples. The results are listed in Supplementary Table S2 and were used for subsequent analyses. Therefore, the resulting fold-change for each gene represents the additive effect of overexpression and KO after controlling for non-specific effects.

Bootstrap methods were employed to assess the expected overlap under the assumption that MEIS1 and MEIS2 share the same downstream genes. Resampling was conducted for each of the six treatment conditions (MEIS1-OE, MEIS1-KO, MEIS2-OE, MEIS2-KO, ctrl-OE, ctrl-KO), and each condition in a bootstrapped sample was constrained to include at least three unique samples. For each bootstrapped sample, differential gene expression analysis was then performed as described above.

The overlap of Differentially Expressed Genes (DEGs) among MEIS1 bootstrapped samples, or among MEIS2 bootstrapped samples was quantified using the Jaccard index J(A, B) = |A ∩ B| / |A ∪ B|, where A and B represent the DEG sets derived from two bootstrap samples. Confidence intervals were estimated using the basic bootstrap interval.

Jaccard indices of expected and observed DEG overlap were then compared using one-sample Z-score tests, based on the normal approximation of the bootstrap variance^116^. The observed-to-expected ratio was computed by dividing the observed Jaccard Index (J) by the mean J of MEIS1 and MEIS2. Results are reported in Table 1.

The transcriptome-wide correlation between MEIS1 and MEIS2 downstream genes was assessed by calculating Pearson’s product-moment correlation coefficient between the Z-scores of differentially expressed genes (DEGs). The confidence interval was estimated by 100 bootstrap sampling. To identify shared and specific downstream genes, the finite mixture of regression models were employed using the flexmix v2.3–18 package. The number of components in the analysis was determined based on the maximum likelihood solution.

The scatterplots in this study were visualized using a smoothed density color representation in R package graphics. The top 100 data points in the low-density regions were also superimposed on the density image.

### Off-target analysis

We used the CRISPOR^39^ web tool (http://crispor.gi.ucsc.edu/, v4.99) to determine potential off-target sites for each of the four guides used in CRISPR/Cas9 mediated KO, and filtered for sites with CFD > 0.1 and intronic or exonic location. The resulting potential off-target genes were then analyzed for differential expression in our RNA-seq data.

### Gene ontology analysis

Gene Ontology (GO) term enrichment was performed using topGO v2.46.0 with the “classic” algorithm and “fisher” statistic at cutoff FDR 0.05 on Gene Ontology Biological Process (GO BP). Pruned significant GO BP terms with gene set size larger than 20 are shown in Fig. 3. Terms are ordered according to hierarchical cluster Ward.D2 criterion (Murtagh & Legendre, 2014). GO terms were pruned from level 4 of the BP hierarchical structure, retaining only the most significant terms when overlapping genes were identical in each branch. Cross-branch redundancy was removed using Wang’s semantic similarity measures in GOSemSim v2.20.0 with a cutoff at 0.7.

### Chromatin immunoprecipitation

Two batches of human neural stem cells (hNSC) were processed in parallel.

Nuclei were isolated by dissociating cells with Accutase (Sigma A6964) and washing with DPBS. Samples were henceforth processed with the truChIP™ Chromatin Shearing Kit (Covaris, PN 520154) according to the manufacturer’s protocol (010179 Rev K, July 2017). Isolated nuclei were sonicated in a Covaris E220 Focused-Ultrasonicator in milliTUBE–1 mL with AFA Fiber, using nuclei from 30 Mio cells per tube with 5% peak intensity power, 140 W, 200 cycles per burst for 2 min. Shearing efficiency was verified by gel electrophoresis. DNA concentration of the sheared chromatin was quantified using Quant-iT™ PicoGreen (Thermo Fisher Scientific, P7589) on a Cytation 3 microplate reader (Biotek).

Sheared chromatin was adjusted to dilution buffer (final concentration 20 mM Tris-Cl, 150 mM NaCl, 10 mM EDTA, 0.1% SDS, 1% Triton X-100, protease inhibitor) in a total volume of 5 ml. Samples were pre-cleared with 10 µl Dynabeads Protein-A beads (Invitrogen ,10001D) per 40 µl chromatin, by rotating for 4 h at 4 °C. Sheared chromatin was then divided, matching 120 µg chromatin per sample. 1.5 µg of anti-MEIS1/2A K830 and 1.5 µg of anti-MEIS1 K844^16^ were added to each sample, incubating for 16 h at 4 °C on a rotator at 30 rpm. 4.1 µl magnetic Dynabeads Protein A beads per sample were washed 3 times in bead blocking solution (0.5% BSA in PBS) and incubated over night on a rotator beside the chromatin samples. Chromatin was centrifuged at 3,000xg for 20 min at 4 °C. Beads were washed 3 times, resuspended in 80 µl (per sample) immunoprecipitation (IP) buffer (50 mM Tris-Cl pH7.5, 150 mM NaCl, 5 mM EDTA, 1% Triton X-100, 0.5% IGEPAL^®^ CA-630) containing protease inhibitor and divided into single tubes. Supernatant from immunoprecipitated chromatin was added to beads, and rotated for 4 h at 4 °C. At 4 °C, beads were then washed 6x in IP Buffer or 5x in IP Buffer & 3x in IP Buffer + 250 mM LiCl (all without inhibitors), and 1x TE buffer pH 8.0. Samples were eluted from beads in 200 µl bead elution buffer (10% SDS, 100 mM NaHCO_3_) for 30 min while shaking at room temperature. The supernatant was mixed with 8 µl 5 M NaCl and de-crosslinked at 65 °C over night. 4 µl RNase A (10 µg/µl) were added and samples incubated for 30 min at 37 °C. Protein was digested by adding 4 µl EDTA, 8 µl 1 M Tris-Cl and 4 µl proteinase K (10 µg/µl) and incubating at 55 °C for 2 h. DNA was then purified using the MinElute PCR purification kit (Qiagen, 28004), with 1 ml Buffer PB and 50 µl 3 M NaOAc (pH 5.2). DNA was quantified using PicoGreen.

Illumina sequencing libraries were generated using the NEBNext Ultra II Library Prep Kit for Illumina (E7645) with NEBNext Multiplex Oligos for Illumina (Dual Index Set1, E7600) according to the manufacturer’s protocol. Size selection was performed after PCR using AMPure XP beads (Beckman Coulter). Due to the high number of large fragments > 1000 bp remaining, additional size selection was performed using a Pippin Prep (Sage Science), using a 2% cassette with size window set to 300–750 bp. Samples were sequenced 36 bp paired-end on an Illumina NextSeq550.

### ChIP-seq analysis

Adapters were trimmed from raw reads using cutadapt (v1.16), without quality-based trimming. Reads were mapped to the human genome assembly GRCh37 (hg19) using bowtie2 (v2.3.0) and samtools (v1.2) at a cutoff of mapq > = 30. PCR duplicates were removed with Picard tools (v 2.15.0). Peaks called by MACS2 in paired-end mode with a P-value cut-off of 0.01 were used as input for IDR analysis^117^ (v.2.0.3), which was used to determine reproducible peaks between the two replicates.

HOMER^49^ (v4.10.4) was used for de-novo motif analysis and enrichment of known motifs with default settings, in 500 bp windows centered on peak summits.

BETA plus^56^ was used to associate MEIS1 target genes (all DEG at FDR < 0.05 and FC > 1.5) with MEIS binding sites at a maximum TSS distance of 1 Mb. To predict the activating and repressive function of MEIS1, and for motif analysis in binding sites associated with MEIS1 target genes, we used all DEG with an FDR cutoff of 0.05.

Pileups used in Fig. 5& Supplementary Figure S6 were generated using the Integrative Genomics Viewer v2.18.4^118^.

## Recources

CRISPR guide sequences.

**Table:** 

guide ID	sgRNA sequence
CRISPRa-MEIS1-g2	TGTGTGTTGCACAGGCGGAG
CRISPRa-MEIS2-g8	GAGTGAGTGTCAGTAGGTGT
hsnt-1	ACGGAGGCTAAGCGTCGCAA
CRISPRKO-MEIS1-g2	GGTGGCCACACGTCACACAG
CRISPRKO-MEIS1-g3	AAAAAGCGTCACAAAAAGCG
CRISPRKO-MEIS2-g2	CCCACTCAGCAGGCACCCCA
CRISPRKO-MEIS2-g5	GTTCTGATGTCAGGATACCA

Plasmid vectors.

**Table:** 

Plasmid name	Source	Identifier
lentiCRISPRv2	Feng Zhang / Addgene	Cat# 52,961
lenti sgRNA(MS2)_puro optimized backbone	Feng Zhang / Addgene	Cat#73,797
lentiMPH v2	Feng Zhang / Addgene	Cat#89,308
lenti dCAS-VP64_Blast	Feng Zhang / Addgene	Cat#61,425
psPAX2	Didier Trono / Addgene	Cat#12,260
pMD2.G	Didier Trono / Addgene	Cat#12,259
pCW57-MCS1-2 A-MCS2	Adam Karpf / Addgene	Cat#71,782

Antibodies.

**Table:** 

Name	Source	Identifier	Application
Anti-MEIS1	Abcam	ab19687	Western blot
Anti-MEIS1	Azcoitia et al., 2005		ChIP-seq
Anti-MEISA	Azcoitia et al., 2005		ChIP-seq, western blot
Anti-MEIS2 AB1	Azcoitia et al., 2005		western blot
Anti-MEIS2 AB2	Sigma-Aldrich	WH0004212M1	Western blot
Anti-GAPDH	Sigma-Aldrich	G8795	Western blot
Anti-β-actin	Abeomics	11–13002	Western blot
HRP anti-rabbit	BioLegend	406,401	Western blot
HRP anti-mouse	Jackson ImmunoResearch	115-036-062	Western blot

## Electronic supplementary material

Below is the link to the electronic supplementary material.

Supplementary Information 1.

Supplementary Information 2.

## Data Availability

ChIP-seq and RNA-seq datasets generated in this study are available from the NCBI Gene Expression Omnibus under the accession numbers GSE271673 and GSE271674. Processed data is available within the paper and its Supplementary Information.
