# Supplementary material for: RLS-associated MEIS transcription factors control distinct processes in human neural stem cells
Source: Sci Rep. 2024 Nov 22;14:28986. doi: 10.1038/s41598-024-80266-9 (PMC11584712; doi:10.1038/s41598-024-80266-9)
Supplement: Supplementary file 1 — Supplementary Information 1. [file 41598_2024_80266_MOESM1_ESM.pdf]

Supplementary information

Title: RLS-associated MEIS transcription factors control distinct processes in human neural stem cells

Authors and Affiliations: Volker Kittke[1,2,3,‡,*], Chen Zhao[1,2,‡], Daniel D. Lam[1,2,4], Philip Harrer[1,2,], Wojciech Krezel[5], Barbara Schormair[1,2,3,†,*], Konrad Oexle[1,2,†,*], Juliane Winkelmann[1,2,3,6,†,*]

Affiliations:

[1] Institute of Neurogenomics, Helmholtz Munich, Neuherberg, Germany

[2] Institute of Human Genetics, Klinikum rechts der Isar, School of Medicine, Technical University of Munich, Munich, Germany

[3] DZPG (German Center for Mental Health), Munich, Germany

[4] (current address) Global Computational Biology & Digital Sciences, Boehringer Ingelheim Pharma GmbH & Co. KG, Biberach an der Riß, Germany

[5] Institut de Génétique et de Biologie Moléculaire et Cellulaire, Illkirch, France

[6] Munich Cluster for Systems Neurology, SyNergy, Munich, Germany

[‡] These authors contributed equally to this work

[†] These authors jointly supervised this work

* Corresponding Authors

Supplementary Tables

Supplemantary Table S1 (.xlsx)
Differential Gene Expression Analysis - individual conditions

Supplementary Table S2 (.xlsx)
Differential Gene Expression Analysis - Contrast Model

Supplementary Table S3 (.xlsx)
Potential off-target sites for CRISPR/Cas9-KO guides

Supplementary Table S4 (.xlsx)
Gene Ontology Analysis of MEIS1 and MEIS2 target genes

Supplementary Table S5 (.xlsx)
MEIS ChIP-seq peaks in hNSC

Supplementary Table S6 (.xlsx)
Direct target gene predictions

Supplementary Figures

Supplementary Figure S1

hNSC at the time of RNA/protein isolation.

Supplementary Figure S2

Protein expression changes of MEIS1 and MEIS2 after CRISPR treatment in hNSC

Supplementary Figure S3:

Gene expression changes by MEIS1 and MEIS2 are correlated

Supplementary Figure S4

GO terms regulated by both MEIS1 and MEIS2

Supplementary Figure S5:

Binding sites of MEIS1/2 in hNSC

Supplementary Figure S6:

MEIS1 Transcription factors directly regulate *MEIS1* and *MEIS2* genes

Supplementary Figure S7

Uncropped western blots

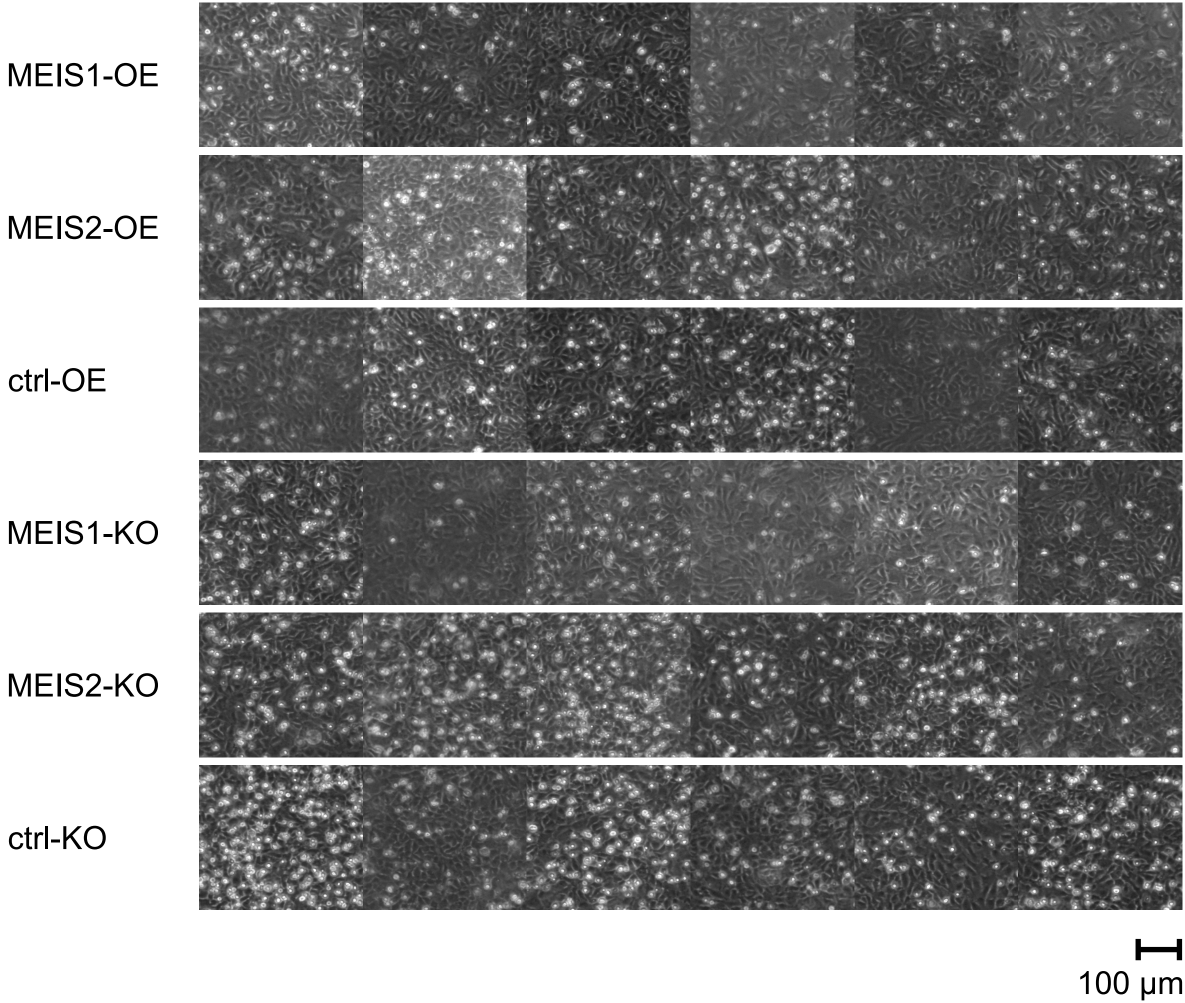

Supplementary Fig S1: hNSC at the time of RNA/protein isolation. Light microscopy images off all samples. Images were recorded 48 h after infection. Images were recorded on an Evos FL digital inverted microscope (Life Technologies) using a 4x objective.

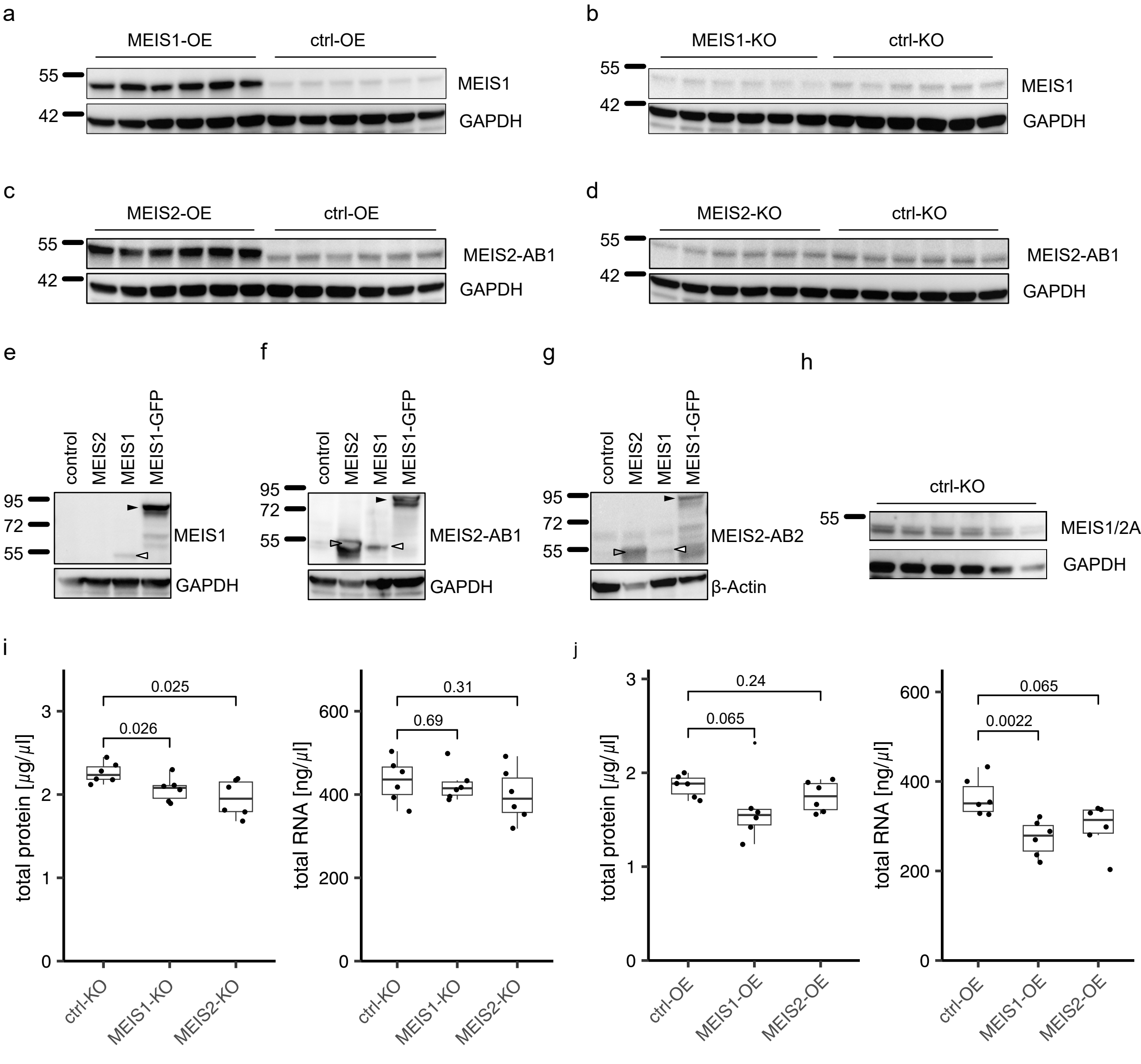

Supplementary Figure S2: Protein expression changes of MEIS1 and MEIS2 after CRISPR treatment in hNSC. **a,b**, MEIS1 protein expression after MEIS1-OE and MEIS1-KO. **c,d,** MEIS2 protein expression after MEIS2-OE and MEIS2-KO. **e-g**, overexpression of MEIS1, MEIS2 and MEIS1-GFP fusion protein in HEK293T cells. Black arrowheads indicate MEIS1-GFP, white arrowheads indicate MEIS1A, grey arrowheads indicate MEIS2A. **h**, detection of MEIS1A and MEIS2A isoforms using anti-MEIS1/2A antibody. **i,j**, total protein and total RNA concentrations isolated for each sample. Conditions are compared against respective controls by Wilcoxon rank sum test, nominal P-values are given for each comparison.

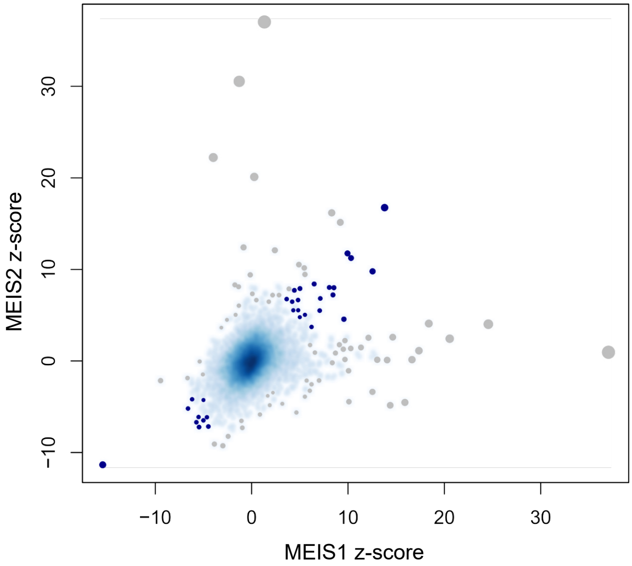

Supplementary Figure S3: Gene expression changes by MEIS1 and MEIS2 are correlated. Z-score smooth scatter plot of MEIS1 and MEIS2 target genes. The x-axis and y-axis represent the Z-scores for the effects on global gene expression by MEIS1 and MEIS2, respectively. Blue and grey dots that are superimposed with the density image represent differential expressed genes that clustered into the middle and extreme slopes in linear mixture model.

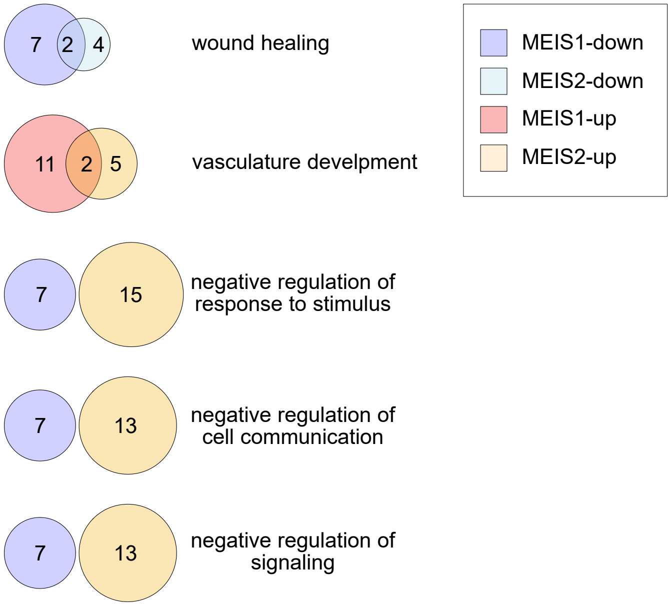

Supplementary Figure S4: GO terms regulated by both MEIS1 and MEIS2. Venn diagram indicates overlap between MEIS1 and MEIS2 target genes in each gene set, that was significantly enriched for both MEIS1 and MEIS2 targets.

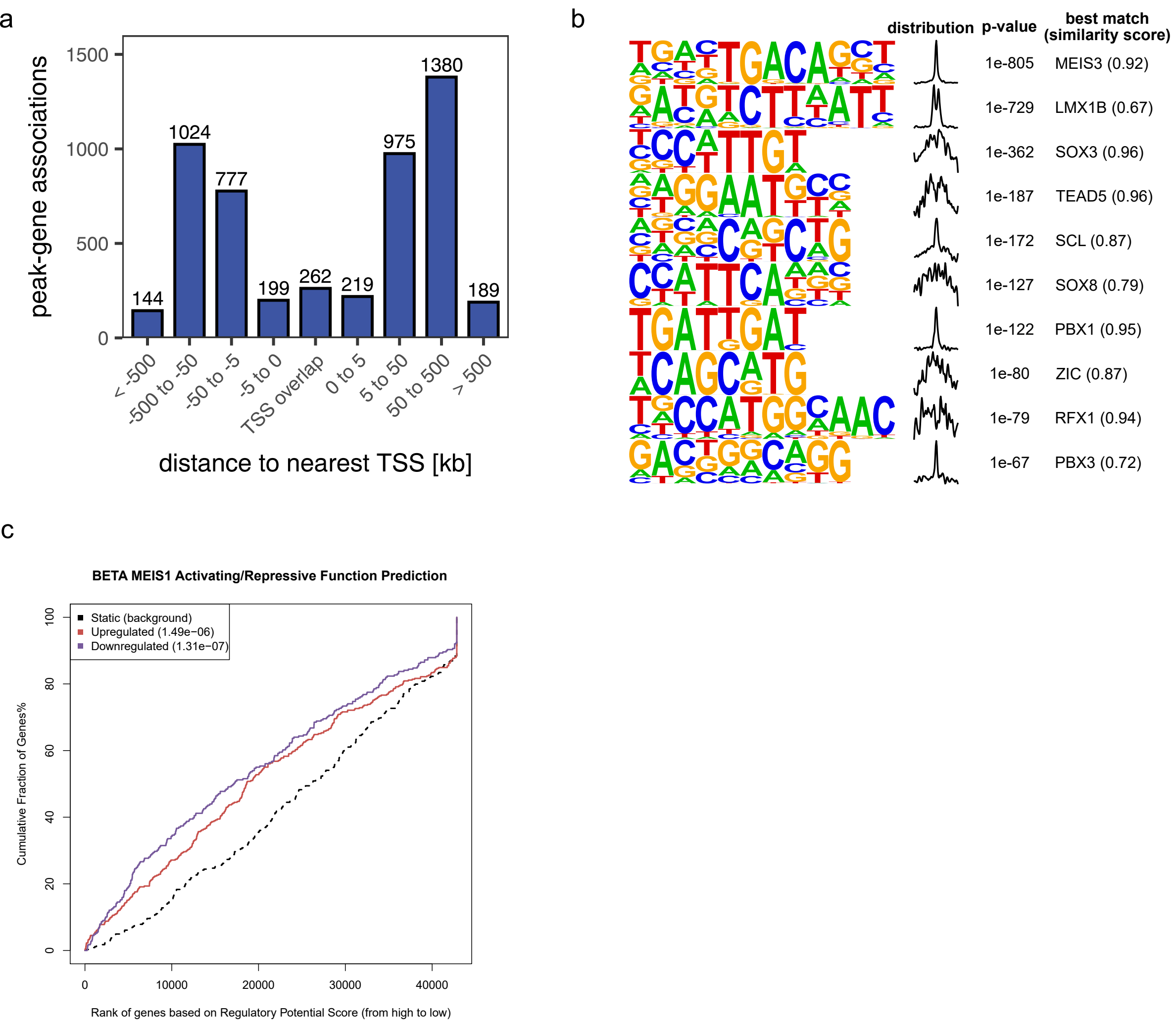

Supplementary Figure S5: Binding sites of MEIS1/2 in hNSC. a, MEIS1 peak distribution relative to the neares TSS. b, Top de-novo enriched motifs within MEIS1 peaks, determined by HOMER. Distribution of motif occurrences across all peaks relative to peak centers **c,** BETA activating/repressive function prediction. The red and the purple lines represent the upregulated and downregulated genes, respectively. The dashed line indicates the non-differentially expressed genes as background. Genes are cumulated by the rank on the basis of the regulatory potential score from high to low. P values that represent the significance of the UP or DOWN group distributions are compared with the NON group by the Kolmogorov-Smirnov test.

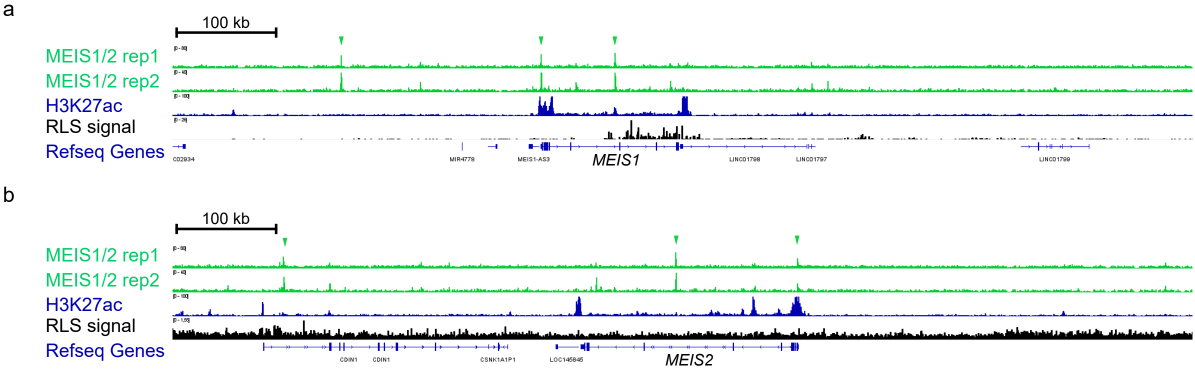

Supplementary Figure S6: MEIS1 Transcription factors directly regulate *MEIS1* and *MEIS2* genes. Binding landscape of MEIS1/2 around **a,** MEIS1 and **b,** MEIS2. H3K27-acetylation data was previously generated in ESC-derived hNSC[60]. RLS SNP association shows Z-scores of GWAS summary statistics for each SNP[4]. Arrowheads indicate called MEIS1/2 peaks.

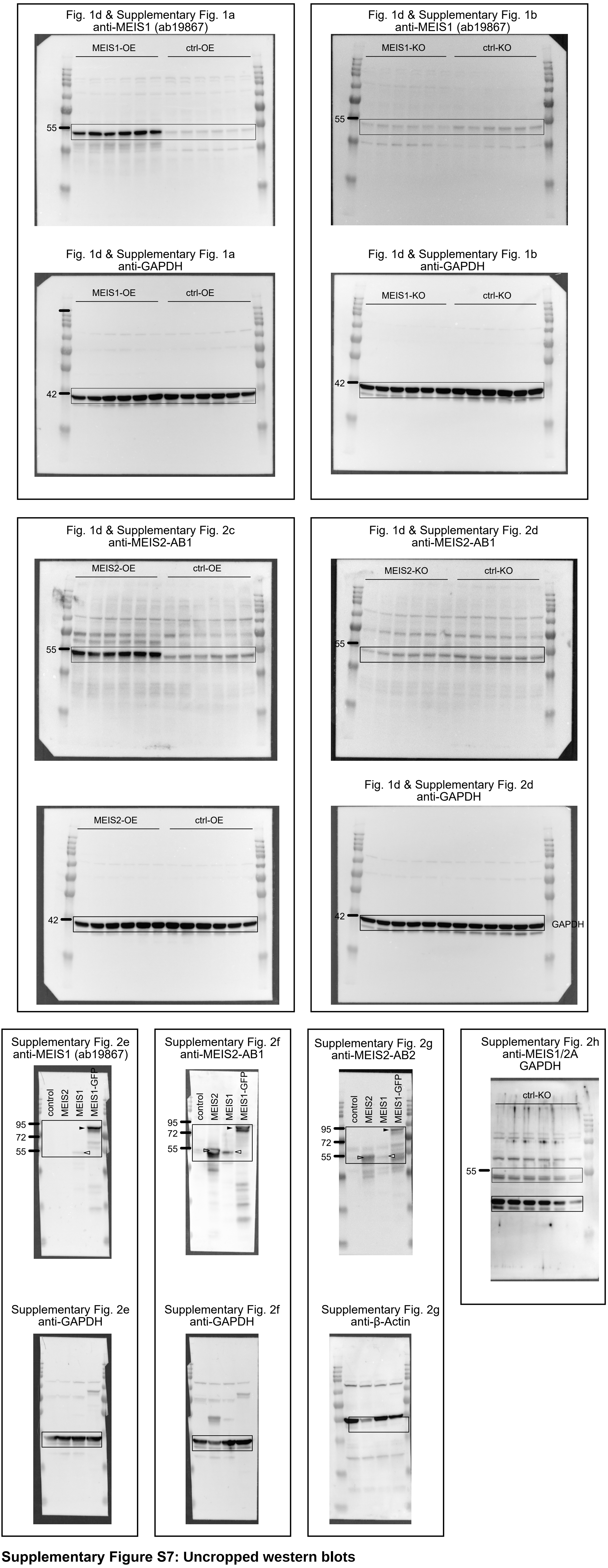

Supplementary Figure S7: Uncropped western blots

ProSieve™ QuadColor™ Protein Marker band sizes [kDa]: 315, 250, 180, 140, 95, 72, 55, 42, 26, 17
